# Real-space anisotropic dielectric response in a multiferroic skyrmion lattice

**DOI:** 10.1038/srep08318

**Published:** 2015-02-09

**Authors:** P. Chu, Y. L. Xie, Y. Zhang, J. P. Chen, D. P. Chen, Z. B. Yan, J. -M. Liu

**Affiliations:** 1 Laboratory of Solid State Microstructures and Innovation Center of Advanced Microstructures, Nanjing University, Nanjing 210093, China; 2 Institute for Quantum Materials, Hubei Polytechnic University, Huangshi 435000, China

## Abstract

A magnetic skyrmion lattice is a microstructure consisting of hexagonally aligned skyrmions. While a skyrmion as a topologically protected carrier of information promises a number of applications, an easily accessible probe of the skyrmion and skyrmion lattice at mesoscopic scale is of significance. It is known that neutron scattering, Lorentz transmission electron microscopy, and spin-resolved STM as effective probes of skyrmions have been established. In this work, we propose that the spatial contour of dielectric permittivity in a skyrmion lattice with ferromagnetic interaction and in-plane (*xy*) Dzyaloshinskii-Moriya (DM) interaction can be used to characterize the skyrmion lattice. The phase field and Monte Carlo simulations are employed to develop the one-to-one correspondence between the magnetic skyrmion lattice and dielectric dipole lattice, both exhibiting the hexagonal symmetry. Under excitation of in-plane electric field in the microwave range, the dielectric permittivity shows the dumbbell-like pattern with the axis perpendicular to the electric field, while it is circle-like for the electric field along the *z*-axis. The dependences of the spatial contour of dielectric permittivity on external magnetic field along the *z*-axis and dielectric frequency dispersion are discussed.

Magnetic skyrmion structure, a topological vortex-like swirling spin order, is now attracting a great deal of interest^1^,^2^. The research interest not only comes from application potentials for next-generation spintronic devices (so-called skytmionics)^4^, but more relies on unexplored physics since a skyrmion is more or less a quasi-particle of specific topological pattern and high stability. Skyrmion may be a unique and topologically protected carrier of charge, magnetic moment, or other favored order parameters and can respond fast to various stimuli of low magnitude. In fact, the major issues of recent intensive investigations on skyrmion and its crystalline form, the so-called skyrmion crystal (SkX), include searching for more SkX materials, exploring microscopic mechanisms for SkX formation, developing techniques for imaging SkX, and manipulating the dynamics of motion among many others.

So far, SkX has been observed mainly in some metallic and semiconducting compounds of chiral B_20_ structure^5^. These compounds include MnSi, FeGe, and Fe_1–x_Co_x_Si etc. Subsequently, the effects of SkX and its dynamics on the transport and magnetism have been emphasized with big interest. The SkX formation in transition metal insulating oxides has not been revealed until very recent year. The Lorentz transmission electron microscopy (LTEM), small angle neutron scattering (SANS), and spin-resolved scanning tunneling microscopy (SR-STM) studies on Cu_2_OSeO_3_ thin films and bulk crystals revealed the existence of SkX^6^,^7^,^8^. This finding allows additional possibilities to correlate the SkX with other physical properties. Typically, due to the multiferroicity found in Cu_2_OSeO_3_, as done in the pioneer work of Seki et al^3^,^7^,^9^,^10^,^11^, one is able to deal with the dielectric/ferroelectric behaviors and spin ordering correlated with SkX as well as its dynamics from various aspects, no need to mention that the dielectric/ferroelectric spectroscopy of SkX in these materials is highly favored too. This is one of the major motivations for the present work.

Our understanding of the microscopic mechanisms for the SkX formation stems from comprehensive knowledge on spin frustration in complex transition metal magnets in which competing interactions make the spin ordering highly degenerate, resulting in incommensurate and noncollinear spin orders, as well as specific spin orders of topological pattern like skyrmion. Theoretically, it is suggested that the competition between the *S_i_*·*S_j_*-like symmetric exchange interaction and the asymmetric *S_i_* × *S_j_*-like Dzyaloshinskii-Moriya (DM) interaction, where *S_i_* is the spin moment at site *i*, is the key ingredient for chiral spin structures, and consequently the inversion symmetry may be broken. In addition, magnetic field *H* plays a substantial role in manipulating the SkX structure and its dynamics. This physics can find its analogy with that for multiferroicity in a lot of complex transition metal oxides. Thus it is not strange to observe the coexisting SkX and multiferroicity in Cu_2_OSeO_3_. One may be in a position to expect more multiferroic materials with SkX.

Based on the above discussion, our major interest is to explore the correlation between the SkX and dielectric/ferroelectric behaviors in a multiferroic system. First, the most concerned multiferroics of magnetically generated ferroelectric polarization are those magnets with noncollinear spin order and strong DM interaction, which are the two necessary ingredients for the SkX too^12^. The intrinsic correlation between the SkX and magnetically induced ferroelectricity is physically reasonable^13^. Second, while magnetic and electric current controls of skyrmion motion have been interested^14^,^15^, electric field control of this motion appears to be more promising for practical applications not only due to the technological competing advantages and low energy cost. If the electric polarization has the same magnetic origin as that the SkX has, a physically accessible approach to electric field control may be no longer a challenge^16^. Third, an easily accessible probing of the SkX, in particular the spatial structure of spin order is still appealing. Although the LTEM, SANS, and SR-STM are powerful for such probing, electrical probing based on dielectric/ferroelectric responses would be more useful and competitive. In fact, recent investigation along this line by Seki *et* *al* on Cu_2_OSeO_3_ represents a substantial forward step^17^,^18^.

It is well known that for those ferroelectrics of noncollinear spin order, the electric polarization can be described by the phenomenological theory proposed by Mostovoy, and the physics has been well understood. For a SkX lattice, the six-fold rotation symmetry and the twofold rotation axes are followed by time reversal symmetry. This implies no macroscopic polarization over the whole SkX lattice. Nevertheless, for each skyrmion in the lattice, the in-plane incommensurate spin order allows a local topological unit of electric dipoles^19^. In corresponding to the SkX lattice, these local units constitute an electrically polarized topological lattice (for convenience, one may call it the dielectric SkX lattice) which can be probed using spatially resolved dielectric spectroscopy. Surely, this dielectric SkX lattice may have slightly different topological pattern if an electric dipole is treated equivalent to a spin. This electric dipole lattice and associated spin SkX lattice together constitute the two components of a multiferroic SkX lattice.

So far no phenomenological model on the correlation between the two components is available. In this work, we start from conventional multiferroic theory on noncollinear spin order induced ferroelectricity and intend to propose a phenomenological model on which the spatially resolved dielectric responses can be investigated. Obviously, such a dielectric response spectrum may provide an attractive roadmap to map the multiferroic SkX lattice into an electrically measurable pattern, so that various dielectric spectroscopy techniques such as piezoresponse force microscopy (PFM)^20^ can be used to detect the SkX and its dynamics. Considering that the dielectric response here mainly comes from the fluctuations of electric dipoles, the frequency spectrum would be in the microwave range (~GHz).

Owing to the complexity of the interactions involved in the SkX lattice, we deal with a simplified Hamiltonian model. We start from a cubic lattice with dimension *L* and with periodic boundary condition so that the open boundary issue can be avoided. We only consider the DM interaction between the spin pairs on the *xy* plane. This assumption implies that the as-generated skyrmion most likely exhibits a cylinder-like pattern along the *z*-axis, similar to the cases reported in Refs. 21, 22. Therefore, one may map the spin and electric dipole structures on the *xy* plane for illustration.

Here, we assume that the ferroelectric (FE) polarization *P* = (*P_x_*, *P_y_*, *P_z_*) in magnetically induced ferroelectrics is a second-order order parameter generated by the primary order parameter, i.e. magnetic moment *M* *= * (*M_x_*, *M_y_*, *M_z_*), via the magnetoelectric (ME) coupling. In a good approximation, the Landau potential for electric dipoles can be written as 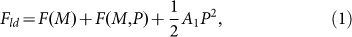
where *F*(*M*) is the energy for magnetic order parameter *M*, *F*(*M*, *P*) stands for the ME coupling^23^, and the last term is the self-energy of *P* with *A*_1_ the energy coefficient. Regarding term *F*(*M*, *P*) in an incommensurate spin lattice, the invariance upon the time reversal and spatial inversion sequence must be maintained. When *M* undergoes a transition from paramagnetic phase to the SkX phase, a nonzero local *P* appears as the consequence of the ME coupling, but this FE order related energy is weak with respect to the magnetic interactions.

Following the Ginzburg-Landau (GL) theory on the SkX lattice, one has^24^: 
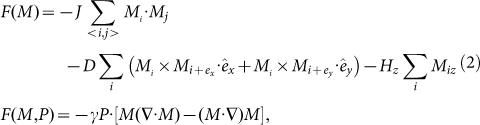
where *J* stands for the ferromagnetic interactions along all the three axes (*x*, *y*, *z*), <…> denotes the nearest-neighbor (NN) pairs, *D* is the DM interaction coefficient which is nonzero only for the *xy*-plane spin-pairs, *γ* is the ME coupling coefficient, *H_z_* is a static weak magnetic field which imposes a weak anisotropy along the *z*-axis. It is noted that the ME term is microscopic mechanism dependent. For example, in multiferroic Cu_2_OSeO_3_, polarization *P* is suggested to come from the *pd*-hybridization mechanism^9^,^25^. Correspondingly, term *F*(*M*, *P*) can be written as 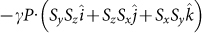 using the symmetry derivation in Ref. 23. Our simulations using this *F*(*M*, *P*) term as the ME term did qualitatively reproduce the main characteristics reported experimentally in Ref. 9 in terms of the spatial distributions of the electric polarization and local charge density (results not shown here). In this work, we focus on a phenomenological model to investigate the spatially patterned dielectric response in a microwave electric field for the SkX lattice. Therefore, we take the ME term proposed by Mostovoy^23^. The results involving various microscopic mechanisms for the ME coupling will be presented elsewhere in future, while the present mechanism seems ot be of generality.

Since the Landau potential *F_ld_* only describes the energy of a single electric dipole, a series of energy terms associated with the interactions between the electric dipoles should be taken into account, referring to the standard GL theory on tetragonal FE lattice^26^. The total energy for this multiferroic lattice (including both the spin and dielectric SkX components) can be written as: 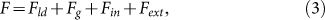
where *F_g_*, *F_in_*, and *F_ex_* are the gradient energy, self-electrostatic energy and the energy induced by external electric field *E_ext_*. For term *F_g_*, the lowest-order expression is: 
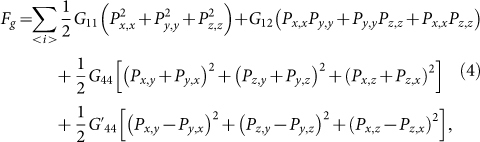
where *P_i,j_* *=* *∂P_i_/∂x_j_* and *G*_11_, *G*_12_, *G*_44_ and *G*′_44_ are the gradient energy coefficients^27^. Terms *F_in_* and *F_ext_* can be written as *F_elec_*: 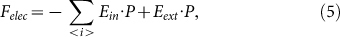
where *E_in_* is the self-electrostatic electric field which is the negative gradient of the electrostatic potential *ϕ*, i.e. *E_in_*(*i*) = −∂*ϕ*/∂*x_i_*, and *E_ext_* the external electric field, respectively. The electrostatic potential is obtained by solving the electrostatic equilibrium equation^28^

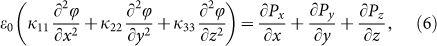
where *κ_ij_* is the background dielectric constants.

To access the dielectric response over frequency domain, we calculate the dielectric permittivity *ε*(*ω*) over a broad range of frequency, which corresponds to the microwave frequency range for real systems since the dielectric permittivity here comes from the electric dipole fluctuations in an *ac*-electric field^29^. The algorithm and details of the calculation procedure are presented in the Method section. The parameters for the simulations are listed in Table 1.

## Results

### Local polarization distribution in SkX lattice

**Figure 1:**
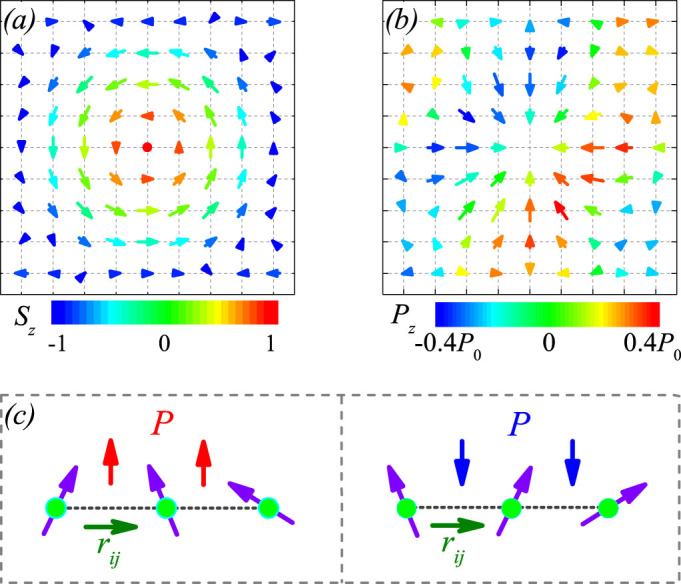
Illustration of a multiferroic skyrmion. (a) Spin configuration with *S_z_* indicated by color, (b) spatial distribution of polarization with *P_z_* indicated by color. (c) Two directions of generated polarization predicted by inverse DM interaction mechanism.

Before presenting the simulated results on the multiferroic SkX lattice, as an example we plot the spatial pattern of a simulated spin skyrmion from the lattice, as shown in Fig. 1(a), where the spin components on the *xy* plane are indicated by arrows and the *z*-axis component is scaled by colors. The simulated pattern is similar to earlier reported skyrmion pattern^30^. The spins on the peripheral sites are anti-parallel to the *z*-axis and the centric spins tend to align along the *z*-axis, whereas on the bridging region are aligned the counter-clockwise chiral spins on the *xy* plane.

The as-generated configuration of electric dipoles for this skyrmion is presented in Fig. 1(b). It is seen that the as-generated electric dipoles are aligned non-collinearly and their magnitudes are spatially inhomogeneous. The total polarization over this unit is zero. This electric dipole configuration is different from the spin skyrmion, but we name it as a dielectric skyrmion simply for convenience. According to the inverse DM interaction mechanism for ferroelectricity, a nonzero electric dipole is described by *P_ij_* = *Ar_ij_* × (*S*_i_ × *S*_j_) where *r_ij_* is the unit vector connecting two neighboring spins *S_i_* and *S_j_*, and *A* is the pre-factor measuring the spin-orbit coupling^31^. Clearly, when *S_i_* and *S_j_* are on the *xy* plane, the generated electric dipole has the maximal magnitude since *r_ij_* is also on the *xy* plane. In Fig. 1(c), a schematic drawing of the electric dipole generation is given, following the inverse DM interaction mechanism.

**Figure 2:**
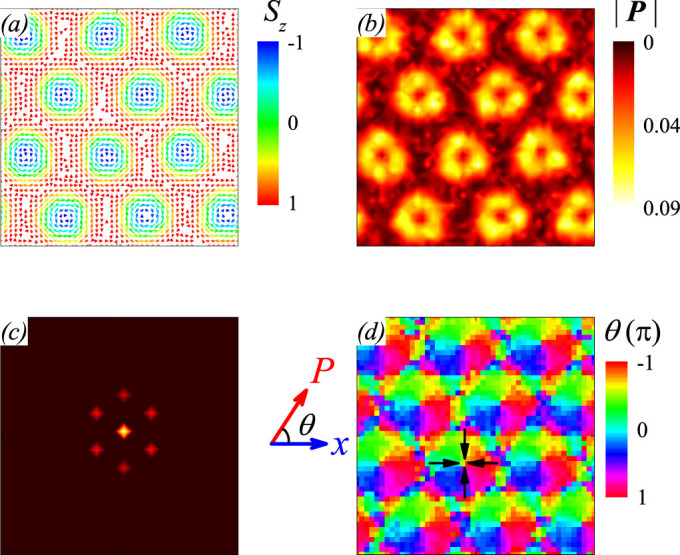
Simulated multiferroic domain structure, (a) spin configuration with *S_z_* indicated by color, (b) spatial distribution of polarization magnitude, (c) Bragg intensity pattern of the spin configuration, and (d) the FE domain structure. Black arrows indicate the direction of dipoles in each domain, magnetic field is along the out-of-plane direction.

Subsequently, we look at the spin SkX lattice and as-generated dielectric SkX lattice, as shown in Fig. 2 for *E_ext_* = 0 and *H_z_* = 0.5(*D*^2^/*J*). As expected, the spin SkX lattice exhibits well-aligned hexagonal symmetry, as shown in Fig. 2(a), which is also identified by the Bragg pattern shown in Fig. 2(c). The simulated dielectric SkX lattice is plotted in Fig. 2(b), showing the hexagonal symmetry too although the electric dipoles are non-collinearly aligned. The orientation and magnitude of electric dipoles in each skyrmion are discussed in Fig. 1 and no detailed discussion is given. For reference, Fig. 2(d) presents the in-plane orientation of the electric dipoles over the whole lattice and a roughly hexagonal symmetry can be seen too. This one-to-one correspondence between the spin and dielectric lattices constitutes the basis for spatially resolved dielectric spectroscopy of the magnetic skyrmion lattice. It should be mentioned that the dielectric SkX lattice, similar to the case of a single dielectric skyrmion, shows no net macroscopic polarization if no electric bias is applied. However, for practical measurements, an electric poling is used and thus a small polarization will be observed, such as *P* ~ 16*μ*C/m^2^ at 5 K for Cu_2_OSeO_3_, as revealed experimentally^3^. Obviously, the as-generated depolarization energy can be safely ignored in comparison with the magnetic interactions.

### SkX dynamic response in ac electric field

**Figure 3:**
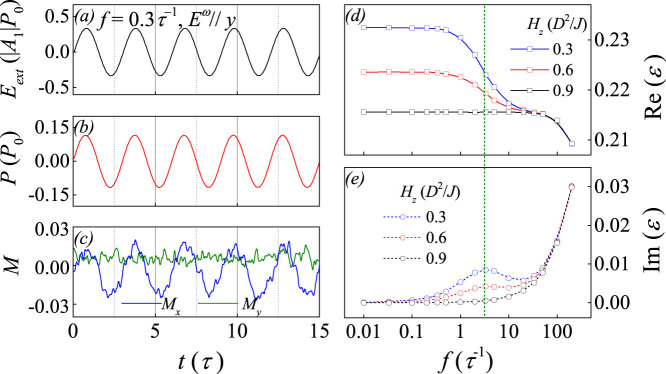
(a) The *ac* electric field *E_ext_* as a function of time *t* at *f* = 0.3*τ*^−1^, (b) the total polarization as a function of time *t*, and (c) the *x* component and *y* component of magnetization as a function of time *t*. Simulated dielectric permittivity spectrum (d) real part and (e) image part around *f* = 1.0*τ*^−1^ given *H_z_* = 0.3, 0.6, and 0.9*D*^2^/*J* along the out-of-plane direction. The *ac* electric filed *E^ω^* is along *y*-axis and *E_0_* *= * 0.5|A_1_|*P*_0_.

The above results appear to be the basis for investigating the dielectric response of the SkX lattice, to be discussed in details below. We simulate the responses of polarization *P* and dielectric permittivity *ε*(*f*) = Re(*ε*) + *i*Im(*ε*) to *ac* electric field *E_ext_* along a given direction in the microwave frequency range. Fig. 3(b) presents the simulated *P* as a function of time *t* for an *E_ext_* along the *y*-axis with *E_0_* = 0.35|*A*_1_|*P*_0_ and *f* = 0.3τ^−1^. The simulated components of magnetization along the *x*-axis and *y*-axis, *M_x_* and *M_y_*, are plotted in Fig. 3(c). The instant response of the *P* is observed. For *E_ext_* along other directions, similar results can be observed. Furthermore, due to the ME coupling described by *F*(*M*, *P*) in Eq.(2), the instant response of *M_x_* is identified too while *M_y_* remains unchanged. Due to the dominated ferromagnetic arrangement along *z*-axis, component *M_z_* is either insensitive to *E_ext_*. This ME coupling effect can also be characterized by the response of the dielectric frequency spectrum to varying magnetic field *H_z_*. In Fig. 3(d) and Fig. 3(e) are plotted respectively the real part Re(*ε*) and imaginary part Im(*ε*) of the dielectric permittivity as a function of frequency *f* at three different *H_z_* (in unit of *D*^2^/*J*). According to the phase diagram in Ref. 32, the spin SkX lattice can be stabilized for 0.23 < *H_z_* < 0.78. In this case, the Re(*ε*) and Im(*ε*) are expected to exhibit remarkable frequency dispersion due to the existence of the dielectric SkX lattice, as confirmed by the data at *H_z_* = 0.3 and 0.6. However, for *H_z_* > 0.78, the ferromagnetic phase replacing the SkX phase is favored, implying the disappearance of magnetically induced electric dipoles and thus dielectric frequency dispersion. Indeed, the data at *H_z_* = 0.9 shows no longer any dispersion. Here it should be mentioned that the high-*f* frequency dispersion at *f* ~ 100*τ*^−1^ is due to the electric field induced electric dipole fluctuations which have nothing to do with the SkX fluctuations resonated at *f* ~ 3*τ*^−1^.

### Dielectric response pattern of SkX lattice

**Figure 4:**
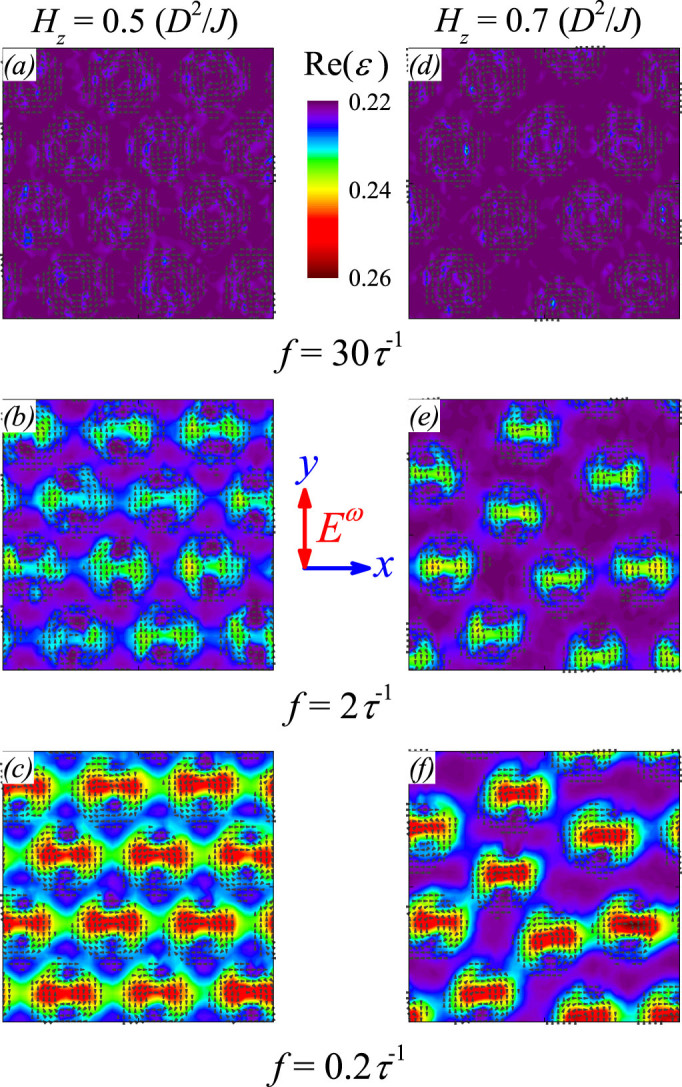
Snapshoted patterns of dielectric permittivity real part Re(*ε*) at different frequency, from top to bottom *f* = 30, 2, and 0.2*τ*^−1^. The left column (a)–(c) is under magnetic field *H_z_* = 0.5*D*^2^/*J*, and right column (d)–(f) *H_z_* = 0.7*D*^2^/*J*. Hereafter, *E_0_* *= * 0.5|A_1_|*P*_0_ and magnetic field *H_z_* is along out-of-plane direction.

Now we investigate the spatial dielectric permittivity patterns of the SkX lattice. The patterns of the real part Re(*ε*) for three different frequencies (three rows) at two magnetic field *H_z_* (two columns) are shown in Fig. 4, given the *E_ext_* along the *y*-axis. Generally, the dielectric real part shows the dumbbell-like pattern with the two-fold symmetry along the *x*-axis, perpendicular to the direction of *E_ext_*. Each dumbbell is centered at one skyrmion center, giving the one-to-one correspondence. The effect of magnetic field *H_z_* on the dielectric response pattern is associated with the stability of the SkX lattice, which is gradually destabilized by increasing *H_z_* from 0.5*D*^2^/*J* to 0.7*D*^2^/*J*, resulting in the broken six-fold symmetry of the SkX lattice^32^. This consequence leads to the dumbbell distortion and gradual suppression of the dielectric response. As expected and shown in Fig. 3d, the dielectric real part is gradually suppressed with increasing frequency *f*, due to the well-known dispersion behavior.

**Figure 5:**
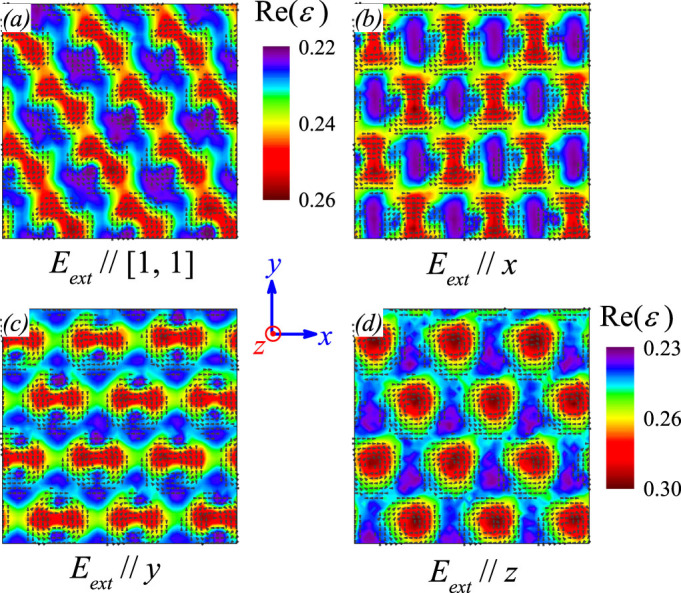
Snapshoted patterns of dielectric permittivity real part Re(*ε*) at constant frequency *f* = 0.2*τ*^−1^ with *E_ext_* along the direction of (a) [1, 1], (b) *x*-axis, (c) *y*-axis, and (d) *z*-axis. Hereafter, *E_0_* *= * 0.5|A_1_|*P*_0_ and magnetic field *H_z_* is along out-of-plane direction.

It is found that the dielectric response pattern can be different if the *E_ext_* is applied along different orientations. For the in-plane *E_ext_*, the dielectric pattern is always dumbbell-like and the axis is perpendicular to the *E_ext_*, as shown by three examples shown in Fig. 5(a)~(c). On the other hand, for the *E_ext_* along the *z*-axis, the dielectric pattern is roughly the same as the skyrmion contour and the six-fold symmetry is reserved, as shown in Fig. 5(d).

## Discussion

To understand the dielectric response patterns upon different *E_ext_*, in particular the dumbbell-like patterns in the cases with in-plane *E_ext_*, one may look at the local electric dipole configuration as shown in Fig. 1(b). Since the dielectric response can be simply viewed as the fluctuations of the electric dipoles against the time-varying electric field, one is able to predict the dielectric response pattern as long as the electric dipole orientation and electric field direction are given. We consider the electric field induced electrostatic energy Δ*F_ele_*: 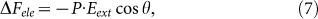
where *θ* is the angle between a local dipole *P* and *E_ext_*. The dependence of Δ*F_ele_* on angle *θ* and the sensitivity can be simply expressed as: 
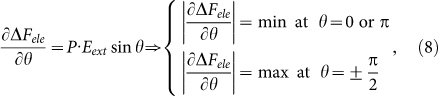
which implies that the sensitivity of Δ*F_ele_* in response to *E_ext_* is much weaker for *P* // ± *E_ext_* than that for *P* ⊥ *E_ext_*, i.e. the dielectric response at *P* ⊥ *E_ext_* is more significant than that at *P* // ± *E_ext_*. Given the in-plane electric dipole configuration shown in Fig. 1(b), this sensitivity argument allows the dumbbell-like two-fold dielectric patterns to align perpendicular to the *E_ext_*, as shown in Fig. 5(a)~(c). However, for *E_ext_* along the *z*-axis, similar prediction can be made and the dielectric pattern is shown in Fig. 5(d). It is seen that only the center region of each skyrmion exhibits relatively large dipole moment and thus the dielectric pattern is circle-like with strong dielectric response on the center region.

To this stage, our simulations reveal that the spin structure of a SkX lattice may be detected by probing the spatial pattern of the dielectric permittivity if this SkX is a multiferroic with sufficiently strong DM interaction. This probing approach is technically convenient and a promising complimentary to spin-sensitive technique such as LTEM and SR-STM. Using a conductive probe to align the microwave beam along the *z*-axis with the in-plane electric field, one is able to detect the spatial resolved dielectric response, which is simple and free of technical challenge.

In summary, we have employed the phenomenological model to simulate the FE domains in multiferroic skyrmion lattice and its dynamic response to *ac* electric field in microwave frequency range. It is found that the SkX lattice can respond to the *ac* electric field and contribute an anisotropic enhancement in the spatial contour of dielectric permittivity. In detail, for the in-plane electric field, a dielectric pattern of dumbbell-like shape with the axis perpendicular to the electric field is shown, while the dielectric pattern is circle-like for the electric field along the z-axis. The present work proposes an alternative approach to probe the SkX through the more accessible dielectric measurement technique.

## Methods

### Simulation methods

Here we have to deal with the dynamic evolution of both spins and electric dipoles simultaneously. We use the Monte Carlo simulation with the annealing algorithm to track the evolution of spin configuration^33^,^34^. Simultaneously, the temporal evolution of the electric dipoles is tracked by solving the time-dependent Ginzburg-Landau (TDGL) equation which takes the following form: 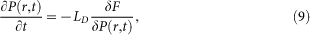
where *t* is time in unit *τ* defined by *τ* ^−1^ = |*A*_1_|*L_D_*, and *L_D_* is the kinetic coefficient. At each step, an evolution of *p_i_* lattice is run in one time step whose scale is decided by kinetic coefficient *L_D_* in Eq.(9). In parallel, the spin lattice evolution is allowed by one Monte Carlo step (*mcs*). We carefully balance the value of *L_D_* for the *P*-evolution and total *mcs* number for the spin configuration evolution, so that the former is much slower than the latter. In this way, the eventual near-equilibrium spin and dipole lattices are obtained.

The parameters for the simulation are listed in Table 1. Dependent on the lattice size, we scale the pitch length of skyrmion *L_s_* according to *D*/*J* = tan(2*π*/*L_s_*)^21^. In our simulation *D* = 0.73~0.50 are chosen to accommodate the lattice size of 36~48, thus the corresponding *L_s_* in these two lattices are 10 and 14. Unfortunately, so far no phenomenological GL theory on multiferroic skyrmion lattice is available. Thus we choose the physical parameters related to polarization for conventional ferroelectrics sharing the similar lattice structure (e.g. BaTiO_3_) as done in our previous works^33^,^34^. For symmetry consideration, this approximation will not induce remarkable difference to our results in qualitative sense. The details of the simulation procedure are described in earlier works^35^,^36^.

### Calculation of dielectric permittivity

We pay particular attention to the dielectric responses of the multiferroic lattice. The dielectric permittivity as a function of *E_ext_* is calculated from the dynamic response of local electric dipoles^33^. For a dielectric system that cannot polarize instantaneously in response to an electric field, the electric polarization as a function of *t* can be described as: 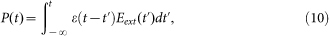
where *P*(*t*) is expressed as the convolution of electric field *E*(*t*) at previous times with time-dependent dielectric permittivity *ε*(*t*) = *ε_r_*(*t*) + *iε_i_*(*t*). Eq.(10) actually defines the dielectric permittivity. This equation can be re-written in the Fourier transform space as: 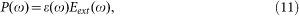
where *P*(*ω*) and *E_ext_*(*ω*) are the Fourier transformations of *P*(*t*) and *E_ext_*(*t*) respectively, and *ω* is the frequency. If a sine electric field *E_ext_*(*t*) * =* *E_0_*sin(*ω*_0_*t*) with *ω*_0_ = 2π*f_0_* and *ω* = 2π*f* is taken, *E_ext_*(*ω*) can be directly written as: 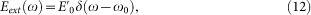
where *E*′_0_ is the coefficient of the Fourier transformation and *ω*_0_ is the frequency of the electric field. Polarization *P*(*ω*) can be calculated by Fourier-transforming the temporal evolution spectrum *P*(*r*, *t*) in Eq.(9). In details, the real-spatial spectrum of *P*(*r*, *t*) at site *r* and its spatial average *P*(*t*) over sufficient number of time periods is calculated by solving Eq.(9). The Fourier-transform frequency spectrum *P*(*r*, *ω*) can be expressed as: 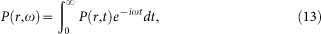
which is used to transform *P*(*r*, *t*) and *P*(*t*) into frequency domain in order to obtain *P*(*r*, *ω*) and *P*(*ω*). Subsequently, one can compute the corresponding dielectric permittivity *ε*(*r, ω*_0_) at site *r* and its spatial average *ε*(*ω*_0_) at frequency *ω*_0_ using Eq.(11).

## Author Contributions

J.M.L. conceived the research project and P.C. did the computations. Y.L.X., Y.Z., J.P.C., D.P.C. and Z.B.Y. commented the modeling and discussed the results. P.C. and J.M.L. wrote the manuscript.

## Figures and Tables

**Table 1:** Physical parameters chosen for the simulation (*τ* ^−1^ = |*A*_1_|*L_D_*)^31^,^36^. All these parameters appear in the dimensionless form

Parameter (unit)	Value	Parameter (unit)	Value	Parameter (unit)	Value
*L*	36~48	A* 1 (|*A*_1_|)	−1.00	J* (*J*/|*A*_1_|*P*^2^_0_)	1.00
D* (*D*/|*A*_1_|*P*^2^_0_)	0.73~0.50	γ* (*γ*/|*A*_1_|*P*_0_)	1.00	T^*^_d_ (|*A*_1_|*P*^2^_0_)	0.001
G^*^_11_ (*G*_11_/*A*^2^_1_|*A*_1_|)	1.60	G* 12 (*G*_12_/*A*^2^_1_|*A*_1_|)	0.00	G^*^_44_ (*G*_44_/*A*^2^_1_|*A*_1_|)	0.80
*G*′^*^_44_ (*G′*_44_/*A*^2^_1_|*A*_1_|)	0.80	κ* (*κ*/|*A*_1_|*P*_0_)	60	τ* (*τ*)	0.0004
